# Pharmacy faculty experiences with student academic entitlement: a multinational study from the Arab world

**DOI:** 10.1186/s12909-024-05402-5

**Published:** 2024-04-28

**Authors:** Dalal Hammoudi Halat, Mervat M. Alsous, Ali Azeez Al-Jumaili, Ahmed Malki, Kawther Khalid Ahmed, Anas Hamad, Rula Darwish, Dixon Thomas, Salma Bukhatwa, Maher Khdour, Nora Alkhudair, Abdullah Ali Al Balushi, Sherif Khalifa, Naser Z. Alsharif, Mohamad Rahal

**Affiliations:** 1 Academic Quality Department, QU Health, Qatar University, Doha, Qatar; 2 Department of Clinical Pharmacy and Pharmacy Practice, Faculty of Pharmacy, Yarmouk University, Irbid, Jordan; 3 University of Baghdad College of Pharmacy, Baghdad, Iraq; 4 College of Pharmacy, Qatar University, Doha, Qatar; 5 Pharmaceutics and Pharmaceutical Technology Department, School of Pharmacy, The University of Jordan, Amman, Jordan; 6 College of Pharmacy, Gulf Medical University, Ajman, United Arab Emirates; 7 Faculty of Pharmacy, Libyan International Medical University, Benghazi, Libya; 8 Al-Quds University College of Pharmacy, Jerusalem, Palestine; 9 Department of Clinical Pharmacy, College of Pharmacy, King Saud University, Riyadh, Saudi Arabia; 10 Oman College of Health Sciences, Pharmacy Program, Muscat, Oman; 11 School of Pharmacy, Lebanese American University, Byblos, Lebanon; 12 School of Pharmacy, Lebanese international University, Beirut, Lebanon

**Keywords:** Academic entitlement, Pharmacy education, Pharmacy faculty, Arab World

## Abstract

Academic Entitlement (AE) is the expectation by students to receive high grades or preferential treatment without significant effort. Exploring AE from faculty perspective has not been investigated in Arab colleges of pharmacy. The aim of this study was to explore experiences and perceptions towards student AE among pharmacy faculty in the Arab World. A cross-sectional, self-administered, anonymous, electronic survey was sent to pharmacy faculty across pharmacy colleges in Arab countries. The survey collected demographic data, an AE measure including 17 items reflecting seven AE components, and faculty perceptions and perceived reasons for AE. A total of 345 responses were collected. The AE level was moderate (46.05 ±7.29), and the highest scores among its components were for customer service expectation (62%) and responsibility avoidance (59%). In multiple linear regression, AE showed positive significant association with faculty in clinical pharmacy departments and those having fewer years of experience. Most common complaints heard by faculty from students were requests to turn in assignments late (90%), while the most common communication issues faculty faced with students were unprofessional verbal communication (58%) and unprofessional messages on social media (57%). Poor admission criteria (40%) and existence of multiple private colleges of pharmacy (37%) were the most common perceived reasons for AE by participating faculty. This study reveals moderate AE experienced by pharmacy faculty in the Arab World, as well as common complaints, communication issues, and AE reasons. In collaboration with other stakeholders, faculty play an important role in indicating expectations from students regarding AE, and research is warranted to check if such interventions reduce AE among pharmacy students.

**Supplementary Information:**

The online version contains supplementary material available at 10.1186/s12909-024-05402-5.

## Background

Across colleges of pharmacy, faculty members frequently witness incidents by students who show improper attitudes towards education, with many acting ungraciously and unprofessionally in the classroom [1, 2]. These actions are based on students’ expectations from faculty members to actively cater them and meet their demands for a convenient education with marginal effort, and include using social media, texting, surfing the web, studying for other courses, coming to class late or departing early, skipping pre-class duties, and failing to successfully attempt team assignments. Most educators have such anecdotal stories to relate about students acting improperly, and it is proposed that a substantial contributing reason to this behavior is Academic Entitlement (AE) [3, 4]. Faculty in touch with millennial students notice that this generation is more technologically savvy, ethnically diverse, and socially connected than any of their student predecessors [5]; yet, the most distinguishing feature of this group, at least in the classroom, may be their AE attitudes [6]. Several definitions of AE have been proposed [4, 7–9], and they reflect beliefs by students that some reward, unjustified by academic achievement, is deserved, like expectation of a higher grade for effort, or that the student should not be faced with failing a major course in their main area of study. Other AE beliefs imply a diminished role for personal responsibility in academic achievement, suggesting that students fail to recognize their personal role in success, but rather consider failure to perform well academically a reflection of the quality of teaching or resources [10]. Academically entitled students believe that it is the instructor’s responsibility to propel them through the class, constantly make them aware of upcoming due-dates, or even track those with difficulties to offer extra assistance. AE also implies unrealistic expectations from students about instructors, such as answering emails and responding to phone messages quickly, or making special, flexible accommodations.. 

Despite the different scales depicted in literature to measure AE among students [4, 11, 12], these scales fall short of measurement of faculty perceptions about this issue, and documented research on AE from the viewpoint of faculty remains scarce. In a study by Stevens and Colleagues [13], although faculty agreed that students are engaged in class, they indicated that specific skills and attitudes needed to perform successfully were not apparent, and were gradually declining. They also believed that students’ skills and work ethic have declined while their sense of entitlement to high grades has risen, coupled to unrealistic expectations in higher education. Although research has started to explore the attributes, behaviors and expectations of students who feel a sense of AE, the impact of AE on the work experiences of teaching staff remains as a gap of knowledge that needs yet to be explored.

For faculty, AE can alter teaching practices; for instance, a study revealed that faculty with a lower academic rank perceived their students as more entitled, with pressures of working towards promotion influencing these professors’ behaviors, making them more accommodating to students, thus impacting the quality of education. Moreover, such encounters with students affect faculty emotional reactions including stress, anxiety, and ability to perform properly [14, 15]. To our knowledge, no previous studies have addressed faculty perceptions and beliefs about AE among pharmacy faculty in the Arab World. As such, the purpose of the current study was to explore perceptions towards AE among pharmacy faculty in different pharmacy colleges in the Arab World, and assess associated factors.

## Methods

### Study design

This study design was a cross-sectional survey of pharmacy faculty and their perceptions towards AE. Data were collected using a self-administered electronic questionnaire posted across pharmacy faculty networks in Arab countries (Lebanon, Iraq, Egypt, Jordan, Palestine, Saudi Arabia, Libya, UAE, Qatar, and Oman). An invitation letter was first sent to pharmacy faculty explaining the purpose of the study and inviting for participation. The electronic survey was administered through Google Forms and its link was open from January 23^rd^ to May 13^th^, 2022. Reminders were sent routinely by co-authors ask their fellow colleagues to respond to the survey. The study follows the Strengthening the Reporting of Observational Studies in Epidemiology (STROBE) checklist [16] for cross-sectional studies. For sample size calculation, the Rao soft sample size calculator (http://www.raosoft.com/samplesize.html). Assuming a confidence level of 90%, a margin of error of 5%, a recruitment rate of 50% and a maximal sample size of 20000 academics, the minimum required sample size was 267.

### Survey instrument

The survey instrument was drafted in English by the co-authors and diligently reviewed, where misleading, confusing, and vague questions were resolved through discussion. The instrument, validity was revised based on Messick’s validity framework where sources of evidence are organized according to content, internal structure, response process, relationship to other variables, and consequences [17]. A preliminary version of the survey was then piloted with 17 faculty from six different countries, and improvements regarding clarity of the survey items were realized. Consequently, some survey items were deleted or revised.

The final survey tool was composed of 30 questions divided into three sections. The first section collected demographic data about the participating faculty including age, gender, country where they work, type of the college of pharmacy (public or private), their highest education level and its country of origin, current rank, department in which they mostly teach courses, years of experience in academia, and whether they were pursuing any post-graduate studies while working at the college of pharmacy.

The second section of the survey included an AE measure in the form of a Likert scale (from 1 =strongly disagree to 5 = strongly agree) based on the study by Jackson and Colleagues [18], but shortened and modified to fit regional academia and terminology, and directed to faculty. The development of this scale involved, besides these original statements, many focus groups, review of other published methods, discussion among authors, and sharing of experience from different perceptions and encounters of faculty in the pharmacy colleges in different countries. Several rounds of revision were conducted for clarity and consistency, to arrive at the current instrument. The final AE measure used in the study included a total of 17 items that correspond to seven components of AE: 2 measuring reward for effort, 2 for accommodation, 2 for responsibility avoidance, 2 for customer orientation, 3 for customer service expectation, 3 for grade haggling, and 3 for general AE. An AE score out of 85 was calculated based upon the summation of the Likert scale responses for these 17 statements.

The third section of the survey addressed faculty perceptions about AE and included three questions that address frequency of student complaints heard by faculty, frequency of communication issues with students, and common reasons faculty perceive for the attitudes of AE in students. The full survey instrument is available in Supplementary file 1.

The survey was voluntary, anonymous, and no incentive was offered to participants. The Research Committee approved the study protocol at the institution where the primary author was at the time of data collection, the Lebanese International University School of Pharmacy (approval number 2021RC-031-LIUSOP). The study was also approved by Qatar University Institutional Review Board (exempt letter number QU-IRB 1668-E/22).

### Data analysis

All analyses were carried out using Statistical Package for the Social Sciences (SPSS®) software version 27, Armonk, NY. Descriptive statistics were used to describe the sample population; the continuous variables were described using mean and standard deviation (SD); categorical variables were described using frequency and percentages and analyzed using Chi-square test. ANOVA and independent-T-Test were used to measure the relationship between the AE score of participants and demographic data, including age, gender, type of college of pharmacy, and qualifications. Multiple linear regression using a stepwise method was then utilized to determine independent predictors of AE scores. Cronbach’s alpha was used to measure the reliability (internal consistency) of the AE scale. The significance level was set at 0.05.

## Results

### Demographics

A total of 345 faculty members responded to the survey from different Arab countries, including Jordan, Lebanon, Iraq, UAE, Qatar, Kingdom Saudi Arabia (KSA), Palestine, Libya, Egypt, and others. The demographic characteristics of the respondents are shown in Table 1. The mean age was 40.41±9.30 years, and the average number of years of experience in academia was 11.50±7.76 years. In this study, females accounted for 57.1% (*n* = 97) of participants. The full demographic data of the participants is available in Supplementary file 2.

**Table 1:** Scores for the seven components of Academic Entitlement (AE)

**AE Component**	**Mean score (%)**	**SD**	**Minimum score**	**Maximum score**
Rewards for effort (Score out of 10)	5.45 (54.5)	1.65	2	11
Accommodation (Score out of 10)	5.81 (58.1)	1.74	2	10
Responsibility avoidance (Score out of 10)	5.93 (59.3)	1.74	2	10
Customer orientation (Score out of 10)	5.81 (58.1)	1.24	2	10
Customer service expectation (Score out of 15)	9.24 (61.6)	2.06	3	14
Grade haggling (Score out of 15)	6.09 (40.6)	2.25	3	3
General AE (Score out of 15)	7.74 (51.6)	2.00	12	13

Scores out of 10 and 15 correspond to AE components with two and three statements respectively

### Academic entitlement score

The AE score for the 17 statements out of 85 was (46.05 ±7.29; mean±SD) with a maximum score of 67.0. The internal consistency (Cronbach’s α) of the AE instrument was 0.70.

The mean score of each component of the seven components of the AE score is shown in Table 1. The reward score was significantly highest in faculty members from pharmacy practice department (5.85 ±1.08; mean±SD) compared to other departments (5.16 ±1.91; mean±SD), *p*-value: <0.001). The grade haggling score was significantly higher in male participants (6.50 ±2.28; mean±SD) compared to females (5.78 ±2.19; mean±SD), *p*-value: 0.003). Other components were not significantly associated with demographic characteristics of participants.

The AE score was significantly associated with several factors, including country of work, source of degree, years of experience, and department of work (pharmacy practice and clinical pharmacy), while other factors like age, gender, highest degree and others, were not associated with the AE score, as shown in Table 2.

**Table 2:** Factors associated with Academic Entitlement (AE) score (*N*=345)

**Factor**	**AE score**	***p*-value**
**Age**		
<40 (*N*=166)	46.25±7.41	0.609
≥40 (*N*=179)	45.84±7.17	
**Gender**		
Male	46.37±8.25	0.491
Female	45.81±6.48	
**Country of work**		
Lebanon	45.19±6.79	0.015*
Iraq	47.76±7.86	
Jordan	44.88±7.71	
Palestine	49.53±4.46	
Libya	48.67±4.32	
Egypt	46.56±6.40	
KSA	44.63±6.43	
UAE	48.00±7.83	
Qatar	41.74±6.57	
Oman	45.03±7.55	
Other	46.05±7.29	
**Highest education level**		
PhD	45.86±7.62	0.608
MSc	46.57±6.91	
BSc pharmacy	46.85±4.18	
PharmD	44.47±7.67	
Resident	43.25±7.36	
Post-doctoral	50.25±4.99	
**Type of college of pharmacy**		
Public university	45.44±6.73	0.081
Private university	46.36±8.07	
Both public and private universities	48.83±5.78	
**Current faculty rank**		
Professor	44.52±7.57	0.544
Associate Professor	44.92±8.33	
Assistant Professor	46.68±7.28	
Lecturer	46.56±7.11	
Adjunct assistant Professor	48.20±4.60	
Other	45.99±7.27	
**Source of degree**		
Arabic University or University in country of residence	47.01±7.35	0.001*
University Europe or USA	44.16±6.99	
University from Asia	47.73±6.90	
**Years of experience**		
≥11 (*N*=162)	45.15±7.40	0.035*
<11 (*N*=182)	46.80±7.10	
**Pursuing postgraduate study**		
Yes	46.27±7.49	0.794
No	46.00±.7.26	
**Department**		
Pharmaceutical Sciences	44.31±7.78	0.022*
Medicinal chemistry and pharmacognosy	46.14±6.72	
Pharmacy practice and clinical pharmacy	46.93±7.10	
Pharmacology and toxicology	45.00±6.99	
Clinical biochemistry and clinical lab sciences	48.61±7.27	
Other	51.40±2.88	

^*^Significant difference (*p*-value < 0.05)

Multiple linear regression, using a stepwise method, yielded a final model that included only two variables that were independently predictive of the AE score, i.e., department (clinical pharmacy) and having fewer years of experience (Table 3).

**Table 3:** Predictors of Academic Entitlement (AE) score using multiple linear regression

**Independent variable**	**B**	**SE**	**Beta**^**a**^	**95% CI**	***p*-value**
Department (Clinical pharmacy)	0.88	0.32	0.15	0.26-1.51	0.006*
Years of experience	-0.11	0.05	s-0.12	-0.21- -0.01	0.029*

*B* regression coefficient, *SE* Standard error associated with the coefficient *B*, ^a^standardized coefficient, *p-value < 0.05

### Frequency of complaints heard by faculty from students

**Fig. 1:**
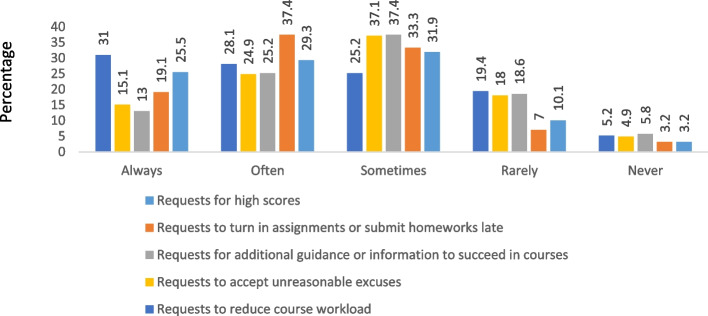
Frequency of complaints heard by faculty from students regarding requests reflecting Academic Entitlement (AE)

The most common complaint reported by participating faculty was requests from students to turn in assignments or submit homework late (89.85%,) followed by requests for high scores (86.66%) (Fig. 1). The later complaint was highly reported by faculty members in the pharmacy practice and clinical pharmacy departments compared to members from other departments (*p*-value = 0.008). Other complaints were not statistically significantly different between faculty members with different demographic characteristics.

### Frequency of communication issues faculty faced with students

**Fig. 2:**
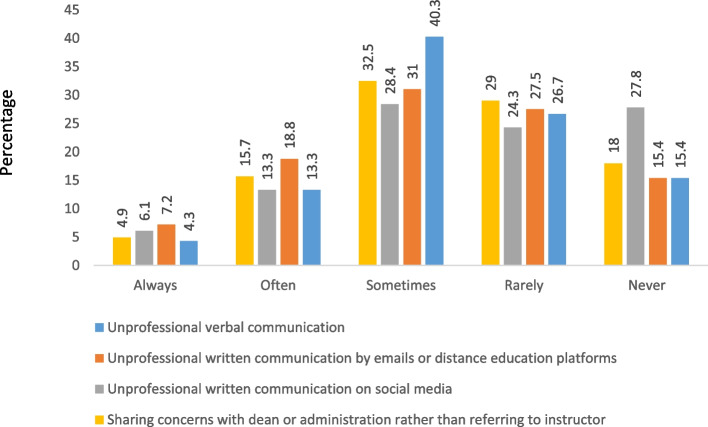
Frequency of communication issues with students reported by faculty

Figure 2 shows a representation of reported communication issues that have been faced with students by faculty, where the most common issue was unprofessional verbal communication, reported by 58.0% of participants, and this was significantly higher among faculty members who have a BSc degree compared to higher academic degrees (*P*-value <0.031).

Also, 57.1% of participants reported that they receive unprofessional written messages on social media, especially those faculty members who got their degree from an Arabic country or country of residence (p-value = 0.006), those who work in KSA (*p*-value <0.001), and those not currently pursuing a postgraduate study.

In addition, the percentage of respondents who claimed that they receive unprofessional emails or written messages on a distance education platform was significantly higher among lecturers compared to other academics with different work ranking (*p*-value = 0.012) and those who work in KSA (*p*-value <0.001).

### Reasons for academic AE among students

Faculty perceived multiple reasons for AE; the results are presented in Table 4. The most common reasons agreed upon were poor admission criteria to colleges of pharmacy (40%) followed by existence of multiple private colleges (37%). The least perceived reason for AE according to the participants was inadequate faculty experience in dealing with students. On the other hand, 47% of respondents disagreed that lack of guidance and monitoring of students from advisors and faculty was a reason for AE attitudes from students. Easy access of students to social media where they broadcast their complaints was agreed upon by 31% of participants as a reason for AE, and was significantly more reported by PhD holders compared to faculty members with other work ranking (*p*-value = 0.032). Moreover, pressure from administration on faculty as a reason for AE was reported by 36% of participants, and was significantly associated with those who work in public universities (*p*-value = 0.036).

**Table 4:** Perceptions of faculty regarding reasons for Academic Entitlement (AE) attitudes by students

**Reason for AE**	**Responses**				
	**Strongly disagree** **N (%)**	**Disagree** **N (%)**	**Neutral** **N (%)**	**Agree** **N (%)**	**Strongly agree** **N (%)**
Pressure from administration on faculty	33 (9.5)	89 (25.8)	99 (28.7)	89 (25.8)	35 (10.1)
Dependence on student evaluation of faculty	17 (4.9)	115 (33.3)	113 (32.8)	77 (22.3)	23 (6.7)
Promotion issues (where teaching excellence and student evaluation are counted in ranking/promotion)	20 (5.8)	122 (35.4)	96 (27.8)	79 (22.9)	28 (8.1)
Inadequate experience of the faculty in dealing with students	21 (6.1)	128 (37.1)	103 (29.9)	80 (23.2)	13 (3.8)
Easy access of students to social media where they broad cast their complaints	35 (10.1)	116 (33.6)	86 (24.9)	60 (17.4)	48 (13.9)
Existence of multiple private colleges pf pharmacy to choose from	29 (8.4)	98 (28.4)	91 (26.4)	91 (26.4)	36 (10.4)
Poor admission criteria at the college of pharmacy	26 (7.5)	105 (30.4)	78 (22.6)	74 (21.5)	62 (18.0)
Lack of guidance and monitoring of students from advisors and faculty	32 (9.3)	129 (37.4)	75 (21.7)	74 (21.5)	35 (10.1)

## Discussion

This study is the first in the Arab world to conduct an analysis of AE perceptions and experience among pharmacy faculty, and the first multinational study addressing this issue among faculty from the Arab countries. The identification of AE characteristics and faculty views is important in the context of how this affects educational outcomes in pharmacy colleges, and what remediating measures are needed to reduce influences of AE on teaching and learning [10]. This is especially important with recent findings of high AE scores among pharmacy students in the Arab World and the negative impact of these scores on student professionalism [19]. Projecting into future pharmacy graduates, this analysis is also essential in another context of research showing positive relationship between AE at college and prospective workplace entitlement [20].

According to our baseline analysis, a moderate score of AE, of approximately 46 out of 85 was obtained upon measuring faculty experiences with pharmacy students’ AE. Literature depicts a variety of tools to measure AE, and although some AE measures were well validated and replicated in studies, others were solely used by the developing researchers [18]. Hence, a good consensus on AE measures and which attributes they capture remains an intriguing area. Examples of well-developed, commonly used AE measures are the 15-item, 2-dimension measure developed by Chowning and Campbell [4] and the 8-item, 5-domain measure developed by Kopp and Colleagues [11]. In 2020, Garg and Colleagues [12] developed a novel measure of AE based the pharmacy student as the customer or the product of education, and linked that measure to student professionalism. The current study used a 17-item instrument extrapolated from a previous study based on a multi-stage effort to establish a measure for AE in a 7-dimension scale, nicely reported along with descriptive statistics and a preliminary evidence of validation [18]. In our study, this original tool was shortened for ease of administration, and tailored to fit faculty in pharmacy colleges in the region, and it showed acceptable internal validity (measured by Cronbach’s α). The 7-component structure of this tool aligns with previous research indicating that AE should be treated as a multidimensional construct rather than a single entity [11, 21]. For instance, students’ expectations of unearned academic success are captured in the components measuring reward for effort, responsibility avoidance and grade haggling. On the other hand, students’ expectations of undeserved academic services are apprehended in customer orientation and customer service expectations components, while those of unrealistic expectations from faculty are captured in the accommodation component. Finally, the general AE component conceptualizes that some students may possess entitled attitudes and behaviors in academia due to entitlement that is likely a stable, broader part of their personality [4]. In other words, this last component relates to psychological entitlement rather than specific AE manifestations, as previously described [7].

Breaking down the AE score into its components, the least AE component score was for grade haggling (41%) and was significantly experienced by male faculty. Whether this means that students perceive their grades as fair, and do not complain about grades or argue for extra points as much as they perform on other components of AE, cannot be fully inferred from our results, but is interesting to explore. On the other hand, the highest mean AE component score was for customer service expectations (62%), followed by responsibility avoidance (59%), then customer orientation (58%). While the statements for customer service expectations include access to professors, and quick professor responses to emails and cell phone calls, those for customer orientation include students’ input into classes and choosing the courses required for their degree. These statements were among the ones showing highest scores in our results, and this observation has been reported elsewhere. Koris and Colleagues [22], showed that students have customer attitudes in areas related to collecting and acting on student feedback, classroom studies, communication with service staff, course design, and teaching methods. Likewise, it was previously shown that while faculty believed in students as the products and not the consumers of pharmacy education, students believed that they are consumers, and accordingly, more likely to be unprofessional in the classroom [12, 23].

As for responsibility avoidance, it was previously reported that it positively correlates with entitlement, grandiosity, and narcissism, and negatively correlates with self-confidence, personal control, need for cognition, agreeableness, and meticulousness [4]. Our results on responsibility avoidance are also in parallel with other findings reported by faculty teaching health professions students who showed AE behaviors related to diminished student responsibility to make up their missed work, among other traits [24]. With such high scores of these AE components perceived by our sample of faculty among their students, counseling students over the importance for looking at their university education from the perspective of a learner rather than a customer, and raising awareness about the importance of responsibility for their personal and professional growth, may be ultimately needed across pharmacy colleges in our region. While the average reward for effort score was 55%, it was significantly higher to be perceived by faculty from pharmacy practice department. Interestingly, student requests for high scores were also significantly more experienced by faculty from these departments. It is widely recognized that knowledge is a requirement for practice experience readiness in pharmacy curricula [25]. Students may perceive that they have invested enough efforts and/or deserve high scores upon moving into practice courses, making them request rewarding benefits and higher scores, as reflected in the experiences of pharmacy practice faculty. This may shed a light on the need to counsel students that practice courses have robust and tangible methods of assessment of student performance that should not be overlooked, and success in these courses will require much student effort and perseverance.

In multiple linear regression, only two variables were independently predictive of a higher AE score, faculty being at a clinical pharmacy department, and having up to 10 years of experience or less. It may be anticipated that in clinical pharmacy courses, faculty supervise students in rotations or monitor them during experiential learning. The expected smaller groups of students and direct interaction with faculty in an environment that is different from the regular classroom may give students opportunities to impose requests on faculty, so the latter perceive them as more entitled. This, however, requires a more focused analysis of various departments to obtain results that are more conclusive. It is possible that the nature of assessment methods in clinical pharmacy departments, being more subjective, may contribute to students' AE behavior. For instance, the use of Objective Structures Clinical Examinations (OSCEs), which involve human assessors and require judgment, could lead to students perceiving that they have more room to negotiate or expect higher grades, as compared to assessments with primarily multiple-choice or short answer questions. Further exploration of the relationship between assessment methods and AE could be a valuable avenue for future research. Regarding years of experience, less experienced faculty might be more accommodating to students, driven by the pressure of working towards higher academic ranks. These faculty would, therefore, perceive students as more entitled. While catering students’ needs may be driven by faculty need for higher teaching evaluations as a requirement for promotion, it indeed affects the quality of educational process and should be well monitored, as reported earlier by Heffernan and Gates [14]. Pharmacy colleges ought to stay vigilant in these scenarios, adopting effective protocols to ensure faculty are not pressured to accommodate irrational or inappropriate student appeals for advantages in ranking or promotion.

According to our faculty experience, inappropriate verbal communication followed by unprofessional written messages on social media were the two most frequently experienced communication issues. These results are in parallel with previous research revealing that AE leads to disruptive and uncivil student behaviors that violate the social norms of academia, such as using unprofessional jargon or rude and demanding e-mails [26, 27]. Regardless of the platform for inappropriate communication, it produces both short- and long-term negative consequences on faculty, such as higher incidence of stress, anxiety, and depression, as well as reduced student retention and higher faculty turnover [28, 29]. Establishing and advocating transparent conduct standards and communication norms, defining explicit consequences for improper behavior, and leadership support to faculty could potentially reduce the occurrence of inappropriate student communication stemming from AE. According to our results, receiving unprofessional emails or written messages on a distance education platform was significantly higher among lecturers compared to other academics. The possible interpretation of this finding might be that lecturers are typically younger in age and less experienced, so students may feel they can approach them easily compared to senior faculty. Such finding remains interesting to further explore. Likewise, KSA was a country were pharmacy faculty reported significantly more of such inappropriate communication, despite national accreditation standards and course syllabi including statements about email etiquette, as described by our co-author from KSA. As such, colleges and schools, as well as individual faculty members, should consider the potential effects of AE, and should be prudent and judicious in how they give in to the demands of students. Faculty might unconsciously be persuaded to cater students to their requests of academic success, and accordingly consent to AE when it spreads and eventually becomes universal in learning environments [3].

Moving on to AE reasons, while only 27% of our participants perceived lack of experience as a good reason for students to be entitled, they pointed out the role of university admission, where most pharmacy colleges in the Arab World are not stringent in selection of students. The next more identified reason was the existence of multiple private colleges of pharmacy. According to previous research [3], the high demand for pharmacy graduates, together with the prompt proliferation of pharmacy colleges and their increased competition for potential students through various marketing and recruitment approaches, all might exacerbate student attitudes of AE. Although faculty at the studied colleges of pharmacy may not have potential influence on recruitment and criteria for student admission, these results are a call for faculty to always send messages to students about the evolution and maturation of the profession into the pharmacist being an essential member of the healthcare team. This entails the need for a full set of skills and characteristics such as problem-solving, critical-thinking, empathy, professionalism, communication, leadership, entrepreneurship, and others [30–32]. Pharmacy education, therefore, should foster and support the growth of students in all these domains, and cannot tolerate requests to meet their demands for an education that looks convenient for them and requires minimal or no effort on their behalf. Rather, faculty should promote healthier and more productive interventions by allowing students to take an active role in their learning instead of relying on entitled behaviors to gain desired outcomes. Meanwhile, faculty can foster an environment where students’ coping with failure or remediation occurs in a way that does not intimidate students’ self-efficacy or self-esteem [33]. Furthermore, AE should be differentiated from proper self-advocacy, the ability of a student to communicate their needs and make decisions about the support needed to achieve them. Research shows that self-advocacy skills are related to students’ academic performance and to successful adaptation to university environment [34]. As such, faculty should show wisdom in temporizing the attitudes of AE versus students asking for reasonable consideration and adjusted deadlines. Concurrently, pharmacy colleges, as well as the full academic landscape in different disciplines, should be supported by the broader university structure that reinforces rules of proper student conduct and acceptable versus unacceptable student behavior.

The strength of this study lies in utilizing a tool that captures different aspects of AE and examining it on a multinational level to obtain data about perceptions of faculty regarding AE among pharmacy students. It serves as an initial assessment for AE from the point of view of pharmacy faculty in Arab colleges of pharmacy and prompts further insightful research in this field. However, our study has limitations. First, the AE tool has not been used before to leverage faculty perceptions, and despite piloting and focused discussions among the research team to come up with a reasonable set of statements on AE perceptions, additional fine tuning and amendments may be still needed. Added to this, comparing the findings with other data for more relevant conclusions is difficult with a new tool. It is anticipated that if the tool is further leveraged and exploited in future research, more purposeful discussions of different findings would give a better vision of how pharmacy faculty experience AE. Moreover, our data include mainly faculty from 10 Arab countries and we may have missed responses from other countries that would give a clearer image of AE perceptions among pharmacy faculty. Also, a more in-depth analysis would have been possible by surveying higher administration personnel at the respective colleges and their perceptions on AE. Since AE is a universal observation in higher education, a better dimension can be given to these findings by exploring faculty in other health education colleges as well.

## Conclusion

In summary, the data presented in this study show a moderate level of student AE perceived by pharmacy faculty in Arab countries and associated factors and reasons. Learning how faculty members view students' AE is crucial, since this can affect how well educators can foster a climate conductive to learning. In order to reduce AE and enhance student learning, pharmacy faculty should reflect on their own teaching practices and how to deal with entitled students, while receiving support from their leadership. The study paves the way for a dialogue between faculty and students as well as between faculty and higher leadership about expectations in pharmacy education. Additionally, decision makers need to be more aware about AE among students and work to minimize such behavior to positively impact student-faculty relationships. To better understand the viewpoints of instructors, additional research in this area might involve other health educators and qualitative study approaches. Finally, it would be beneficial to investigate the efficacy of interventions that reduce or mitigate AE among pharmacy students.

### Supplementary Information

**Supplementary Material 1.**

**Supplementary Material 2.**

## Data Availability

The datasets generated and/or analyzed during the current study are available from the corresponding author on reasonable request.
