# Supplementary material for: Pharmacy faculty experiences with student academic entitlement: a multinational study from the Arab world
Source: BMC Med Educ. 2024 Apr 28;24:470. doi: 10.1186/s12909-024-05402-5 (PMC11057156; doi:10.1186/s12909-024-05402-5)
Supplement: Supplementary file 1 — Supplementary Material 1. [file 12909_2024_5402_MOESM1_ESM.pdf]

Supplementary file 1: Survey instrument

This supplementary file includes the full survey instrument used in this study.

Academic Entitlement Faculty Survey Instrument

Dear Pharmacy Faculty,

Academic entitlement is the unreasonable expectation that students should receive positive academic outcomes independent of performance. In colleges of pharmacy, such expectations can take the bachelor/PharmD students' focus away from the patient to themselves, affecting the quality of education, competency of the future pharmacist, and ultimately, patient care. Entitled students also are a source of pressure on faculty and may alter the pharmacy education experience.

The survey below aims at exploring experience of pharmacy faculty in the Arab World towards academic entitlement. We thank you in advance for helping the research team in filling this survey. The survey takes about 5 minutes to fill and is completely voluntary. You may choose to stop completing the survey at any time. Please note that all survey responses will remain anonymous and confidential, and shall be used for research purposes only.

<u>Section A: Demographic faculty data</u>

1. **Age [open-ended]**
2. **Gender:**
   - Male
   - Female
3. **Country of work:**
   - Lebanon
   - Iraq
   - Jordan
   - Qatar
   - Syria
   - Yemen
   - Palestine
   - Egypt
   - Algeria
   - Morocco
   - Tunisia
   - Libya
   - Sudan
   - Somalia
   - Mauritania
   - KSA
   - UAE
   - Kuwait
   - Oman

- Bahrain
- Comoros
- Djibouti

4.Type of college of pharmacy:

- Public
- Private

5. Highest education level:

- PhD
- MSc
- PharmD
- BS Pharm[1]
- Post-doctoral fellowship
- Residency (PGY1 or PGY2)

6. Current rank[2]:

- Instructor
- Teaching assistant
- Laboratory assistant
- Demonstrator
- Preceptor
- Lecturer
- Assistant professor
- Associate professor
- Professor

7. From where did you get your highest degree?

- Arabic University
- Western (e.g Europe or North America) university
- University in Asia or Africa

8. Are you currently a post-graduate student while at the same time working at a college of pharmacy?

- Yes
- No

9. Years of experience in pharmacy education [open ended].

10. In which department do you mainly teach courses[3]?

- Pharmaceutical sciences

---

[1] BPharm holders are employed in some colleges of pharmacy as full-time lab assistants or teaching assistants.

[2] Different terminology for ranks was used to fit the descriptions used at colleges of pharmacy in the Arab countries.

[3] The departments were based upon structure of most colleges of pharmacy in the Arab countries.

- Pharmacy practice/ clinical pharmacy
- Medicinal chemistry and pharmacognosy
- Pharmacology/toxicology
- Clinical biochemistry and clinical lab sciences
- Others

Section B: Academic entitlement scale

Please rate the following 17 statements on a scale from 1 (strongly disagree) to 5 (strongly agree).

- **1 strongly disagree**
- **2 disagree**
- **3 neither agree nor disagree**
- **4 agree**
- **5 strongly agree**

Reward for Effort

11. Even if students do not perform well, they should get a good grade if they worked hard throughout the course.
12. A student should never fail an assignment he/she had put effort into.

Accommodation

13. Professors should change course rules and schedule (e.g exams and assignment deadlines) for students.
14. The test date should be moved (postponed) if students are not prepared.

Responsibility Avoidance

15. A student is not motivated to put effort into group work, because another group member will end up doing the work.
16. In group assignments, a student should receive the same grade as the other group members regardless of his/her level of effort.

Customer Orientation

17. Students deserve to have more input in how their classes are taught.
18. Students should be able to choose courses required for their degree.

Customer Service Expectation

19. Students should be able to access/reach out to their professors whenever they need help.
20. Professors should respond to students' questions via email, classroom platform, or phone messages within 30 minutes.
21. Students should have their instructor’s cell phone number to contact him or her if they need help.

Grade Haggling

22. Students always deserve a higher grade than they are given, making it necessary to argue for extra points.
23. Students should complain to professors to get the grade they want.
24. Students should complain to the department head or the college dean to get the grade they want.

General Academic Entitlement

25. Students are worthy of more praise from professors for their hard work.
26. Students demand the best grades because they deserve them.
27. Students deserve more excellent grades than they actually receive.

__Section C:__ **Faculty perceptions regarding academic entitlement**

28. **Please indicate which of the following complaints have you heard from students (Never, rarely, sometimes, often, always).**

- Requests for higher scores
- Requests to turn in assignments or submit homeworks late
- Request for additional guidance or information to succeed in courses
- Requests to accept unreasonable excuses
- Requests to reduce course workload

29. **Which of the following communication issues have you faced with your students? (Never, rarely, sometimes, often, always)**.

- Unprofessional verbal communication
- Unprofessional written communication by emails or distance education platforms
- Unprofessional written communication on social media
- Sharing concerns with dean or administration rather than referring to instructor

30. **Which of the following do you believe can enhance student entitlement attitudes? [On a scale from 1 (strongly disagree) to 5 (strongly agree)].**

- Pressure from administration on faculty
- Dependence on student evaluation of faculty
- Promotion issues (where teaching excellence and student evaluation are counted in ranking/promotion)
- Inadequate experience of faculty in dealing with students
- Easy access of students to social media where they broadcast their complaints
- Existence of multiple private colleges of pharmacy to choose from
- Poor admission criteria at the college of pharmacy
- Lack of guidance and monitoring from advisors and faculty
