# Supplementary material for: Pharmacy faculty experiences with student academic entitlement: a multinational study from the Arab world
Source: BMC Med Educ. 2024 Apr 28;24:470. doi: 10.1186/s12909-024-05402-5 (PMC11057156; doi:10.1186/s12909-024-05402-5)
Supplement: Supplementary file 2 — Supplementary Material 2. [file 12909_2024_5402_MOESM2_ESM.pdf]

Supplementary file 2:

This supplementary file includes detailed demographic characteristics of participants in this study.

Participant's demographic data (N=345)

Demographics	N	(%)
**Gender**		
• Male	148	42.90
• Female	197	57.10
**Age (years)**		
Mean±SD	40.41±9.30	
**Years of experience in academia**		
Mean±SD	11.50±7.76	
**Highest education level**		
• BSc in pharmacy	13	3.77
• Pharm (D)	19	5.51
• MSc	101	29.28
• PhD	204	59.13
• Other	8	2.32
**Current rank (**N=343)		
• Instructor	30	8.70
• Lecturer	101	29.28
• Assistant professor	76	22.03
• Associate professor	65	18.84
• Professor	42	12.17
• Other	29	8.41
**Department**		
• Pharmaceutical sciences	90	26.09
• Pharmacy practice/ clinical pharmacy	144	41.74
• Medicinal chemistry and pharmacognosy	44	12.54
• Pharmacology/toxicology	44	12.74
• Clinical biochemistry and clinical lab sciences	18	5.22
• Other	5	1.45
**Type of the college of pharmacy**		
• Public	180	52.17
• Private	141	40.87
• Both public and private	24	7.00
**Source of degree**		
• Arabic university	164	47.54
• University from Asia or Africa	52	15.08
• University from Europe or USA	129	37.39
**Pursuing postgraduate study**		
• Yes	63	18.26
• No	282	81.74
**Country of work**		
• Iraq	79	22.90
• Jordan	74	21.45
• Lebanon	32	9.28
• Oman	30	8.70
• UAE	22	6.38
• KSA	38	11.01
• Egypt	16	4.64
• Palestine	17	4.93
• Qatar	19	5.51

• Libya	15	4.35
• Other	3	0.87
